# Risk Factors with Porcelain Laminate Veneers Experienced during Cementation: A Review

**DOI:** 10.3390/ma16144932

**Published:** 2023-07-10

**Authors:** André Assaf, Shereen S. Azer, Abdo Sfeir, Nadin Al-Haj Husain, Mutlu Özcan

**Affiliations:** 1 Faculty of Dentistry, Beirut Arab University, Beirut 11072809, Lebanon; 2 Division of Restorative and Prosthetic Dentisry, College of Dentistry, The Ohio State University, Columbus, OH 43210, USA; azer.1@osu.edu; 3 Independent Researcher, Beirut, Lebanon; abdo.sfeir89@gmail.com; 4 Division of Dental Biomaterials, Clinic for Reconstructive Dentistry, Center for Dental Medicine, University of Zurich, 8032 Zurich, Switzerland; nalhaj88@gmail.com (N.A.-H.H.); mutluozcan@hotmail.com (M.Ö.); 5 Department of Reconstructive Dentistry and Gerodontology, School of Dental Medicine, University of Bern, 3010 Bern, Switzerland

**Keywords:** adhesion, cementation, ceramics, color, dental materials, porcelain laminate veneers, prosthodontics

## Abstract

The clinical success of porcelain laminate veneers (PLVs) depends on many clinical and technical factors, from planning to execution, among which adhesive cementation is of significant importance. This procedure carries many risk factors if not optimally executed. The objective of this study was to document the clinical parameters affecting successful cementation procedures with a focus on the adhesive strength, integrity, and esthetics of the PLVs. A literature search was conducted through MEDLINE, complemented by a hand search using predefined keywords. Articles published in English between 1995 and 2023 were selected. According to this review, the success and longevity of PLVs rely in great part on the implementation of a precise cementation technique, starting from field isolation, adequate materials selection for adhesion, proper manipulation of the materials, the seating of the veneers, polymerization, and elimination of the excess cement. Several clinical steps performed before cementation, including treatment planning, preparation, impression, and adequate choice of the restorative material, could affect the quality of cementation. Scientific evidence suggests careful implementation of this process to achieve predictable outcomes with PLVs. The short- and long-term clinical success of adhesively luted PLVs is tributary to a deep understanding of the materials used and the implementation of clinical protocols. It is also contingent upon all the previous steps from case selection, treatment planning, and execution until and after the cementation.

## 1. Introduction

A porcelain laminate veneer (PLV) is defined as bonded ceramic restoration that restores the facial, incisal, and part of the proximal surfaces of teeth requiring esthetic restoration (GPT-9). The clinical applications of PLVs cover a broad range of indications for esthetic reconstruction, including diastemas, malalignment, discolorations, fractures, and wear [1]. Several ceramic options are available for PLVs, such as lithium disilicate (LDS), feldspathic porcelain, leucite-reinforced feldspathic porcelain, and LDS reinforced with zirconia [2]. However, some practitioners still prefer feldspathic ceramics for their excellent esthetic properties despite their lower mechanical characteristics and the highly time-consuming and sensitive laboratory and clinical procedures [3,4]. 

Recently, interest in zirconia has been seen due to its favorable mechanical and biological properties. While the lack of translucency in the first generations hindered their use in the esthetic zone, translucency was considerably improved, and ultra-translucent multilayered shaded blocks were introduced [5,6]. However, the multitude of parameters affecting the optical outcome in thin shells limits the use of zirconia translucent blocks to cases with a need for increased thicknesses [7,8]. Moreover, zirconia is not etchable due to the absence of a vitreous phase, while glass-based ceramics are etched with hydrofluoric acid, followed by silanization for a reliable bond to resin-based cement. If bonded to enamel, the weak ceramic structure changes to a mechanically resistant adhesive porcelain veneer complex, particularly feldspathic materials [2,9].

A variety of fabrication methods are available for PLVs; they can be stacked, pressed, or milled via CAD/CAM procedures. Stacking technics are dedicated to the feldspathic materials, which can be built either on a platinum foil or on a refractory die. Lithium disilicates can either be pressed or milled via computer numerical control machining. In a more general sense, any type of glass-based restoration can be digitally generated. 

**Figure 1:**
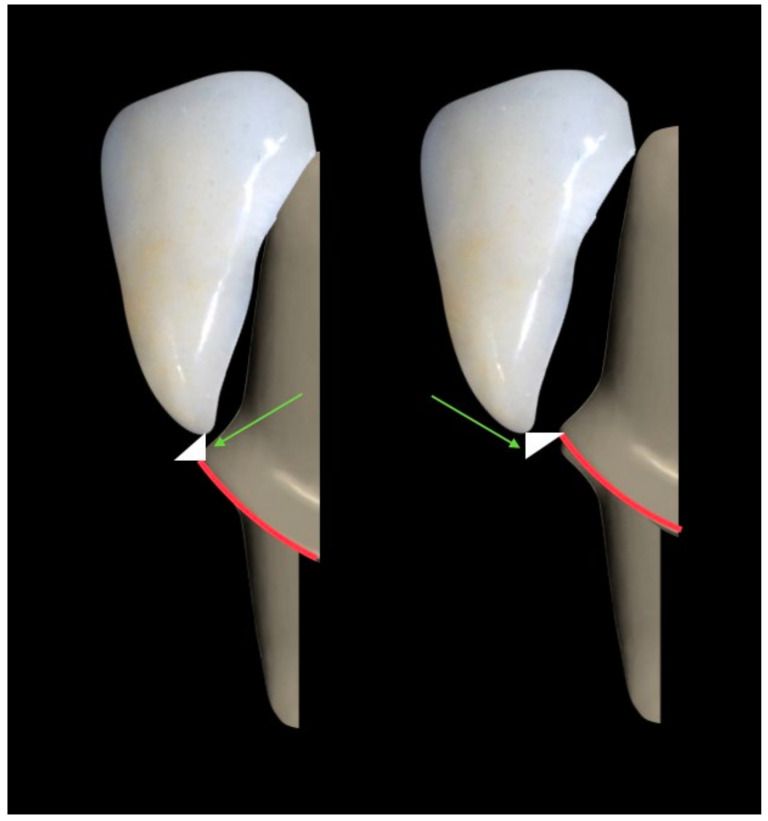
Vertical marginal gap in the case of under-extended and over-extended veneers (green arrow).

**Figure 2:**
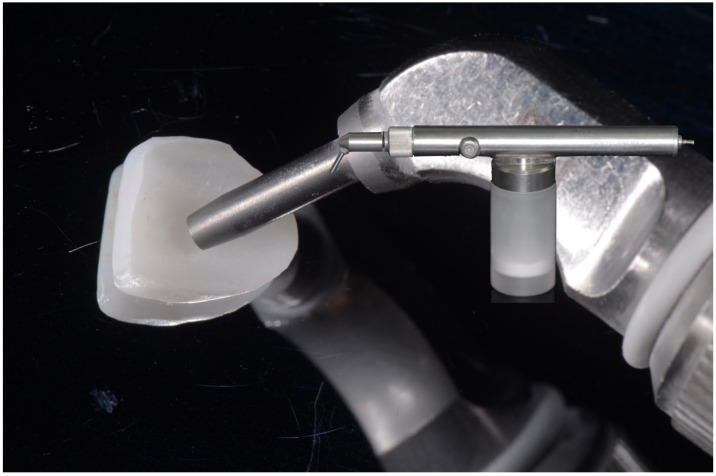
Air-abrasion of porcelain laminate veneer using aluminum oxide particles.

The marginal adaptation of PLVs has been extensively studied. A better vertical marginal fit was reported for platinum foil veneers compared to veneers made with the refractory die technique [10], with a mean vertical marginal gap, defined as the vertical distance between the finish line of the prepared tooth and the margins of the fabricated veneers, was estimated 187 µm versus 242 µm, respectively [11] (Figure 1). These observations are in line with those of Sim and Ibbetson [12] (60 versus 290 µm) and Wall et al. [13] (74 versus 132 µm). However, smaller marginal gap widths were reported, a finding that seems incongruous, considering that the platinum foil occupies 25 µm. According to Lim and Ironside [14], air-abrasion with aluminum oxide particles with the aim of divesting may cause an inadvertent abrasion of the delicate inner feldspathic porcelain surface and accounts for larger marginal discrepancies observed with the refractory die technique (114 µm with sandblasting versus 97 µm without sandblasting) (Figure 2) [10].

In vitro studies of the marginal adaptation of lithium disilicate PLVs reported a wide range of values. Stappert et al. (2007) [15] observed a marginal discrepancy between 43–58 μm. Yuce et al. (2019) [16], comparing the adaptation of heat-pressed and CAD/CAM PLVs after a 2-year follow time, found a range between 295 and 314.98 μm, which was still considered clinically satisfactory.

High success and survival rates are seen in short- and long-term clinical investigations of PLVs’ performance [17,18,19]. In a study conducted by Pneumans et al. in 2004, feldspathic ceramic veneers demonstrated a relatively low rate of secondary caries (≤2%), a high incidence of microleakage (up to 28%), and debonding and fractures (up to 14%) [10]. A high survival rate of 94.4% was reported in a retrospective study with a mean observation time of 5.7 years evaluating 182 anterior porcelain PLVs (feldspathic or pressed ceramic, Empress type) in two different private practices [20]. Similar survival rates of 97.5% were observed in a 7-year follow-up study. Three recorded failures included two endodontic complications (1.7%) at 4 and 5 years, and one secondary caries (0.8%) was detected after 6 years of observation [21].

The fractures of PLV restorations seem to constitute the majority of the observed failures. They might be related to the type of supporting tooth substrate. When cemented on enamel, adhesive failures rarely occur, and adhesive bond strength (BS) can exceed the cohesive strength of the enamel itself. Marginal defects and subsequent microleakage can also be seen, mainly when the margin ends in existing direct composite restorations [1,22].

The influence of location on PLVs’ survival has also been studied, showing a significantly higher rate with maxillary PLVs compared to mandibular ones. This might be attributed to wider surfaces for bonding and, therefore, less risk of failure after long-term use [23]. However, the clinical performance of PLVs has shown a high level of variability. This may be due to the impact of various parameters, such as differences in tooth preparation, type of adhesive agent, adhesion quality, type of supporting substrate, whether enamel, dentin, or restorative material, and marginal adaptation. The proven success can be attributed to a set of well-performed procedures, including (1) case selection, (2) preparation design, (2) proper selection of ceramics for use, (3) proper cementation material and technique, and (4) proper maintenance [3]. The improvements in the adhesive bonding techniques and materials, as well as the clinically acceptable levels of fit with the new ceramics, also support a better clinical outcome. The favorable biological response to the use of dental adhesives and resin-based cement for bonding the PLVs to the vital tooth structure is related to the minimal invasiveness of the preparation procedure aiming at maximal preservation of enamel and avoiding large or deep dentin surface exposure. However, despite controversial results concerning the effect of light polymerization techniques on the degree of conversion, the amount of residual monomer remains a factor to consider biologically, as well as for the retention of the restoration [24]. Nowadays, the adhesive system classification is based on the stages in clinical usages called total-etched and self-etch adhesive methods. Some of them are not polymerized but instead degraded and separated from resin, forming free radicals with proactive agents in induction of toxicity. Furthermore, methacryloyloxy-dodecyl-pyridinium bromide, as an important part of adhesive resins, has been shown to trigger toxicity at high concentrations.

In addition, defective polymerization of free resin monomers and dissolutions with saliva or food intake within the first 24 h can give rise to cytotoxic effects on the pulp tissue. Further cytotoxic effects of adhesive resins are immunosuppression, mild-to-severe inflammation of pulp tissue, and apoptotic cell death.

The purpose of the present review is to relate the scientific evidence deriving from in vitro and clinical studies to the practical context of cementation and underline the impact of the previous clinical procedures on its outcome. The authors explore the risks of complications related to bonding procedures and the post-cementation events such as debonding, marginal discoloration, luting resin dissolution, change in color, incomplete seating, incomplete elimination of excesses and/or fracture or chipping, which may be detrimental for the survival of PLVs.

## 2. Methodology

A wide search was conducted through MEDLINE, complemented with a hand search using the following keywords: porcelain laminate veneers; cementation; dental restoration failure; ceramics; color. Articles published in English between 1995 and 2023 were selected.

## 3. Ceramic Material Used for PLVs

For long-term esthetic improvement of anterior teeth with laminate veneers, two main types of ceramic material are indicated for their translucency, high color stability, potential use in small thicknesses, and capacity of reliable bonding to tooth structure: sintered feldspathic porcelain; and pressable reinforced glass-based ceramic. Both types can also be milled or printed through digital modeling and fabrication, which permit less labor-intensive and time-saving manufacturing procedures [25]. For subtractive production, ceramic blocks are currently available in a wide range of translucencies and shades, whether monochrome or as polychromatic blocks [26]. The material presents a uniform quality, free from internal defects, and the automation allows a reduction in production costs and standardization of the manufacturing processes [3] (see Table 1). Relative translucency of the material is one main property that conditions the esthetic success of the treatment, with multiple contributing factors, which include particle size, particle density, refractive index, porosity, shade, and thickness. The ratio of crystal content-to-glass phase component controls translucency to a certain extent: the more crystalline the microstructure, the more opaquer the ceramic will appear, and the glassier the microstructure, the more translucent the ceramic [3,26]. Veneer restorations with resin composite material still suffer from limited longevity as they remain susceptible to discoloration, wear, and marginal fractures [27]. Indirect hybrid ceramics have a reduced optical matching capacity of the neighboring structures [26]. Based on the treatment goal of being as conservative as possible, the first choice will always be either feldspathic porcelain or glass-matrix ceramics. They showed long-term survival rates of about 80.1%–100% in less than 5 years [28]. Moreover, the production of veneers became even easier with CAD/CAM fabrication. The ceramic blocks are available in a wide range of translucencies and shades, whether monochrome or polychromatic blocks [26]. The material presents a uniform quality, free from internal defects, and the automation allows for the reduction in production costs and standardization of the manufacturing processes (Table 1) [3].

### 3.1. Feldspathic Veneers

Feldspathic veneers are created with layering glass-based (silicon dioxide) powder and liquid materials, followed by a firing cycle to create the final morphology and shade of the restoration. While the crystal phase within the material contributes to the optical properties, the feldspathic veneers demonstrate high translucency due to the prevalence of the glass phase (55% to 70%), which makes them among the most translucent and most esthetic ceramic materials [29]. However, the low flexural strength, usually from 60 to 70 MPa, explains the increased susceptibility to fracture under mechanical stress. Therefore, a good bond, in combination with a stiffer tooth substructure (enamel), is essential to reinforcing the restoration. With this material, it is possible to have a thickness of less than 0.5 mm, with or without preparation in the enamel.

### 3.2. Reinforced Glass-Based Ceramics

**Figure 3:**
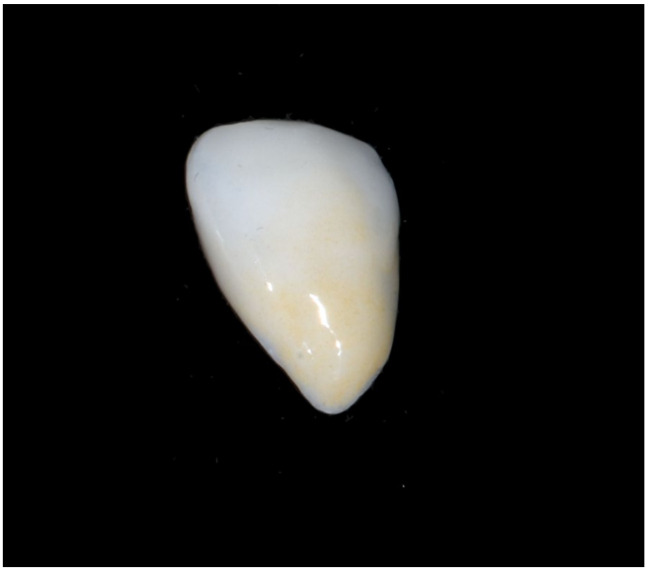
Lithium disilicate veneer.

When increased strength is needed, reinforced ceramics are indicated. The glass matrix is infiltrated by micron-sized crystals of leucite or lithium disilicate, creating a highly filled glass matrix. The microstructure is, therefore, similar to that of powder porcelains, i.e., acid-sensitive and translucent even with the higher crystalline content; this is due to the relatively low refractive index of the crystals. The interaction of the crystals and glassy matrix explains why the optical effects, such as opalescence, color, and opacity, are improved. The size, shape, and number of these crystals also affect the flexural strength. Finer crystals generally produce stronger materials. Currently, leucite and lithium disilicate restorations are fabricated either via heat-pressing techniques or CAD-CAM milling procedures. Compared to stacked feldspathic ceramics, they are less porous (Figure 3) [26].

The ceramics reinforced with lithium disilicate are true glass ceramics, with a crystal content increased to approximately 70% and crystal size refined to improve flexural strength. The material is translucent enough. It presents a variety of shades and can subsequently either receive staining characterization or be veneered with special porcelain. The presence of sufficient room to achieve the desired esthetics is necessary with these materials. Thickness must be at least 0.8 mm, except at marginal areas where they can gradually thin down to approximately 0.3 mm. Because of increased strength and toughness, it can be used in clinical situations with flexure risk factors [3,26]. A new generation of high-strength CAD/CAM ceramics introduced in 2012 is represented by zirconia-reinforced lithium silicate (ZLS). A tetragonal zirconia filler (10% of weight) is added in order to improve its mechanical properties, and the lithium silicate crystals are 4–8 times smaller than those of previous lithium disilicate materials [30].

### 3.3. High-Translucent Zirconia 

Recently, monolithic zirconia has been used for PLVs. In order to achieve adequate translucency, the microstructure of conventional zirconia was modified. Alumina content weight was reduced from 0.5–1.0% to 0.11 to 0.26%, and yttria concentration was raised from 3% to 5–12%. Furthermore, a certain amount of zirconia in the tetragonal phase was replaced by cubic zirconia, thus enabling a more uniform transmission of light through zirconia. Porosity and grain size also affect translucency. Despite the fact that zirconia is generally less translucent than glass ceramics, high translucency can be observed for thinner translucent zirconia, specifically about 0.3 mm [6]. However, few case reports in the literature have demonstrated desirable final shades. It is noteworthy to mention that, regardless of stump shade and cement, thicker restorations generally result in lower values, with the high value being bright white and the low value being dark gray. From a mechanical point of view, its significantly higher flexural strength, confirmed in clinical and in vitro studies, can be regarded as the only main advantage by allowing a much less critical try-in and cementation compared to feldspathic or ultra-thin conventional glass–ceramic veneers [6,30]. However, the possibility of debonding due to less effective adhesion to resin cement represents the greatest shortcoming of this material [30]. Layering the zirconia core with cosmetic porcelain to improve esthetics carries the concern of a high chipping rate of up to 24% after 3 years of use, leading to the switch to monolithic applications.

## 4. Type of Tooth Substrate

The current guidelines underline the importance of maintaining a maximum of tooth structure during preparation. The survival rate of PLVs is higher when the bordering tissue type is an intact enamel [23] since bonding to enamel has been proven to be more efficient and reliable than to dentin [22,31,32,33]. However, the 0.5 mm reduction advocated by some scholars would cause dentin exposure in the cervical region labially and proximally [34]. Based on the study of Ferrari et al. (1992) [31], the amount of preparation should have a range of values from 0.3 mm on the cervical third to reach gradually 0.7 mm on the incisal third. Otherwise, the clinicians might face situations where little enamel or no enamel is left. Although current dentin bonding agents (DBA) are reliable materials, bonding to dentin generates a weaker bond leading to more debonding events compared with enamel, particularly when the veneer restoration is submitted to high occlusal loads [32,35]. Moreover, the absence of the rigid outer shell of enamel is responsible for higher tooth flexure and, consequently, for a higher rate of veneer debonding or fracture [36,37,38]. Hence, with teeth presenting enamel alterations, such as localized enamel malformations and hypoplastic enamel, or in case of over-preparations leading to total facial enamel removal, a significant increase in tensile stresses is created in enamel at the palatal fossa due to the increased flexure of the dentin core [37,39].

## 5. Type of Preparation

The relationship between preparation geometry and service longevity is not fully understood in the literature. It remains one of the most controversial topics concerning the fracture strength of PLVs. Preparation designs can be divided into two major groups, whether with or without incisal overlap [40]. Most clinicians prefer to cover the incisal edge, commonly known as butt–joint design. Others extend the preparation with a chamfer finish line on the palatal or the lingual surface [23]. Albanesi et al. (2016) [41] conducted a meta-analysis to evaluate the performance of ceramic veneers with different preparation designs. The reported estimations of survival rates, defined as the absence of clinical complications (irreparable fractures or debonding), varied between 88% for PLVs with incisal coverage and 91% for those without incisal coverage. These results differed from those reported by Smales et al. (2004) [42], who observed cumulative survival estimates of 95.8% for veneers with incisal porcelain coverage and 85.5% for those without incisal coverage. Interestingly, the meta-analysis of Hong et al. (2017) [43] revealed no statistical differences between any types of overlap, butt–joint, and window-type preparations. Chai et al. (2018) [40] found that butt–joint preparations are superior to incisal overlap with palatal chamfer in terms of veneer resistance to fracture. When the preparation at the proximal area has to be extended lingually, preparing a proximal chamfer becomes clinically challenging and, at the same time, less conservative [44]. A proximal slice is, therefore, advised in certain clinical situations, as in cases of diastema or when a composite filling falls within the preparation outline. Tooth wrapping occurs, a design commonly known as a three-quarter or full veneer preparation. An improved BS of feldspathic veneers was found with three-quarter preparations compared to incisal overlapped preparations [38]. Conversely, it was observed that the marginal adaptation of laminate veneers fabricated on a full veneer prep was poorer compared with minimal wrapping designs. Additionally, a difficult extrusion of excess resin cement during the cementation procedure was observed. It results in incomplete seating, a thicker cement at the margins, and, therefore, more vulnerability to water sorption, polymerization shrinkage, wear, and microleakage. Concomitantly, the cement layer might not be uniform, which can increase the maximum shear stresses in the cement at the bonding surface to values exceeding the BS [35].

Finishing the preparation surface presents a debatable issue regarding its effect on bond quality. Alavi et al. [33] reported in 2007 that leaving the tooth surface unfinished, whether enamel or dentin, can improve the micromechanical retention and consequently slightly improve the BS. On the same line, with no preparation designs, such as when the patient refuses any tooth reduction or when the treatment is additive (e.g., for worn teeth, trauma, fracture, etc.), grinding with a diamond bur or intra-oral air abrasion of the tooth surface is advised [45].

## 6. Type of Substrate vs. type of Restorative Materials

The type of underlying tooth substrate can have a significant effect on the overall survival rate. Understanding its characteristics is crucial for a reasonable selection of ceramic material. Knowing it is one key point for success; the restorative material is chosen according to the tooth substrate to be replaced. From a biomimetic point of view, any removed or missing tooth structure should be substituted with a material of similar mechanical, physical, optical, or biological behavior [37].

The request for less-invasive treatments and higher levels of esthetics have enhanced the indication of feldspathic porcelain. They are excellent substitutes for enamel. It is possible to have a thickness of less than 0.5 mm, with adequate behavior as long as it is bonded to enamel, whether prepared or not [3,32]. However, when flexure risk factors are involved, glass ceramics are recommended due to their increased strength and toughness. They are also indicated where there is more working room to achieve the desired esthetics. These materials are efficient for bonding even if less than 50% of the enamel remains; however, at the margin, at least 30% of the enamel should be present [3].

## 7. Tooth Surface Treatment Prior to Bonding 

### 7.1. Removal of Temporary Cement

In veneer cases, the provisional restorations are fixed either via a cement-free tack-cure technique or by using eugenol-free, calcium hydroxide-based, or photo-polymerized provisional cement. According to several authors, the cleanliness of the tooth surface prior to final cementation can affect the final bonding of the PLVs, particularly to dentin [46,47].

Different cleaning protocols have been tested: prophy cup and flour pumice; dental explorer and air–water spray; cotton pellet and chlorhexidine gluconate; and cleaning bur (e.g., Opticlean) [46,48]. They were compared to prepared tooth surfaces left without temporary cement for effect on the shear bond strength of the bonded PLVs.

Regardless of the cleaning technique, the quality of the permanent bonding was affected when cement or adhesive was not completely eliminated. Tooth surface etching and hybridization would, therefore, be compromised, with the eventual impaired fit of the PLV in extreme cases (see Table 1) [40,49,50,51].

With photo-polymerized provisional cement, particularly when dentin is exposed, temporary cement plugs the orifice of the dentinal tubules. Consequently, resin tags within the dentin tissues cannot be developed well for a reliable bonding [40,52], a finding previously reported by Sarac et al. [52], who observed that the lowest bond strength was obtained with a rotary instrument and a cleaning bur (e.g., Opticlean). Conversely, the study of Zortuk et al. [53] showed no differences in the shear (SBS) values of ceramic discs to dentin surfaces cleaned with a dental explorer, pumice, cleaning bur, and Er:YAG. 

### 7.2. Effect of Tooth Surface Etching

To etch means to produce a retentive surface [54]. This action can be achieved either with the application of an etchant, usually an acidic demineralizing agent, or via the use of lasers. When acidic etching is used, a 37% phosphoric acid agent is applied for a certain duration to the tooth surface and then rinsed abundantly with water. If no dentin is exposed, any type of adhesive can provide an efficient bond. When dentin is exposed, and due to its tubular structure and higher organic composition compared to enamel, the effect of DBA is difficult to control. Phosphoric-acid etching of dentin is generally considered too aggressive. On the one hand, over-etching can lead to denaturation of the collagen fibers, compromising the hybrid layer integrity, especially when monomers do not completely penetrate or do not completely polymerize. This zone could be susceptible to continuous degradation. On the other hand, over-drying the surface after etching and rinsing carries the risk of collapse of the collagen fiber network. Moreover, air drying might increase the movement of dentinal fluids, expressed as pain by sensory fibers at the pulp wall and defined as hyper- or post-operative sensitivity [55,56].

In order to ensure durable bonding on dentin, Van Meerbeek et al. [57] advocated treating enamel and dentin tissue with separate bonding methods. Enamel margins are selectively etched with 37% phosphoric acid, followed by the use of a two-step self-etch adhesive. Such adhesives should contain mild functional monomers with a high chemical affinity to hydroxyapatite [3,57,58,59]. However, a certain risk exists with this technique, related to the spread of the phosphoric acid to the dentin either at its application or during the rinsing procedure. If this occurs, it results in a reduction in the sensitivity prevention advantages [59].

According to some studies, temporization has a negative effect on the quality of bonding to exposed dentin surfaces. Moreover, the impression procedure is capable of over-compressing the collagen fiber network, therefore weakening the BS at the cementation. To overcome these adverse effects, a self-etch DBA treatment of the dentin, known as immediate dentin sealing, is recommended directly after the completion of tooth preparation. It also has the advantage of preventing bacterial invasion and hypersensitivity during interim treatment [23,60,61,62,63].

As an alternative to acid etching, the use of the laser on dental surfaces has been studied by many authors [64]. Controversies exist regarding the effect of its use on BS. However, a recent study showed similar results of SBS when followed by a conventional total-etch bonding protocol. SBS was found to be lower when used with self-adhesive resin cement [65]. Based on this, laser application in dentistry might be considered with a reserve in terms of its effect on bonding. 

### 7.3. Ceramic Surface Conditioning

**Figure 4:**
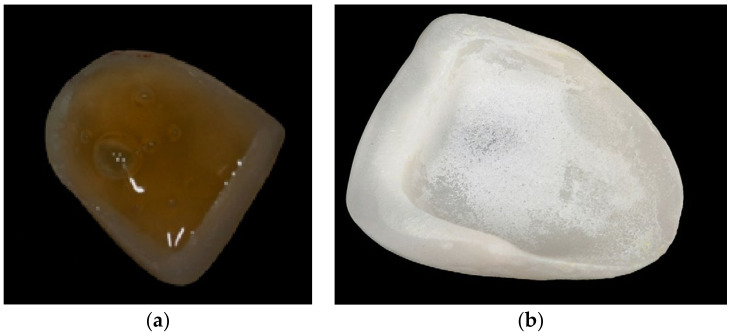
(**a**,**b**): Lithium disilicate veneer (**a**) during and (**b**) after etching. After etching, precipitates are present on the porcelain laminate veneer surface.

Glass-based ceramics are characterized by being easily etchable with hydrofluoric acid (HF), with the aim of roughening the surface and creating porosities that will contribute to a micromechanical bond with the resin cement (Figure 4a,b). This treatment must be followed by a thorough rinsing and application of a coupling agent, known as ceramic primer or silane, prior to the cementation [66,67,68].

### 7.4. Etching the Ceramic Intaglio Surface

An additional advantage of acid conditioning with HF is its efficiency in removing superficial defects and rounding off the remaining flaw tips. This leads to a reduction in stress concentrators and an increase in the overall flexural strength of the bonded veneer. However, etchant concentration and application time are critical to the success of the restorations [69]. They are both specific for each type of ceramic material (see Table 2) [3]. 

Prolonging the etching process or the use of a higher HF acid concentration results in possible weakening of the structure, as demonstrated by biaxial flexural strength tests [70]. Extending etching time carries the additional risk of frosty white surface deposit formation at the etched porcelain surface. It consists of a water-insoluble crystalline residue/salt mix, tenaciously adhering and difficult to remove. It is considered a potential contaminant that could adversely affect the porcelain’s physical properties and/or BS to luting materials. It is, therefore, essential to remove prior to silanization. Different regimens of elimination of crystalline residues have been suggested, with different complexities and efficiencies. Alex G. (2008) [70] reported that air/water spraying of the porcelain intaglio surface was ineffective, even if vigorously executed. The same author found that rubbing with acetone or alcohol was only slightly more effective and that ultrasonication of the etched restorations in ethanol solution for 5 min is usually, but not always, effective in removing any residue. Sometimes, light brushing with a toothbrush and/or longer ultrasonication times are also required [71]. Magne et al., in 2005, used ultrasonication in distilled water for at least 5 min [62]. Later, they advocated the application of 37% phosphoric acid for 1 min, followed by rinsing with water for 20 sec and ultrasonic bath immersion [72]. 

### 7.5. Priming the Intaglio Ceramic Surface

A coupling agent applied to the etched surface has the capacity to link the organic resin-based materials to inorganic metal oxides and glass fillers. However, it is necessary to hydrolyze the silane with acetic acid. It allows the conversion of the 3-methoxy (-OCH_3_) groups located at one end of the molecule to -OH groups, capable of reacting with similar groups present on the surface of the porcelain. In single-bottle systems, the pre-hydrolyzed silane chains have the tendency to react with one another, forming high-molecular-weight oligomers (polysiloxanes). They can function as lubricants, potentially decreasing BS to porcelain [70]. Two-bottle systems consist of an unhydrolyzed silane/ethanol solution in one container and an acetic acid/water solution in the other. Shelf life is longer and is preferable since hydrolysis is controlled by the operator at the bench. However, it is still unclear how much time is required for an acceptable degree of hydrolysis, although some studies recommend leaving the two components on a porcelain surface for a waiting period of 5 min [73].

No matter which system is used, the chemical reaction with the silica component of the ceramic results in covalent bonds through condensation polymerization leading to loss of water. Alcohol and water need to be eliminated from the surface prior to the bonding procedure in order to optimize BS to luting composite. Warm/dry air-drying for 60 s is very effective, and by “heating up” the substrate, the reaction rate would be accelerated, with molecular interactions becoming more frequent, and more chemical bonds could develop [74]. However, excessive application of silane could induce the bonding of consecutive silane layers to each other and create an unnecessarily thick and intrinsically weak coating layer prone to cohesive failure. A shiny surface on the porcelain after drying could be an indication of excessive silane deposition. In such a case, the surface must be sandblasted under low pressure, re-etched with HF, cleaned with ethanol in an ultrasonic, dried, and the silane reapplied. A properly silane-treated porcelain veneer visually appears essentially the same as before silanization, i.e., matte/dull finish (see Table 3) [70,75].

The silane coating is typically formed of three layers, with only the innermost one being hydrolytically stable. Because it is not possible to clinically control the application of only this layer, the removal of the intermediate and outer layers becomes essential for coupling with the resin cement. Many methods exist for the elimination of the excess of silane thickness (see Table 4) [67,76,77,78]. 

Some surface treatments, such as sandblasting (SB) and laser application (Er;YAG laser) aiming at ceramic surface conditioning, might inadvertently reduce the translucency of the PLVs. These treatments might have an even greater effect in this regard when the PLV is particularly thin, whereas HF etching does not seem to affect the translucency parameter (TP) values [79].

The existence of old composite filling at the margin of the PLVs might be a concern in terms of the quality of bonding. Marginal defects are often noticed with laminate veneers ending in existing direct composite restorations [21]. Generally speaking, BS is dependent on the unconverted covalent double bonds (C=C), which can contribute to the adhesion of the luting cement to existing composite restorations. When existing composite restorations have been pre-conditioned, no statistically significant difference between aged and non-aged existing resin composites has been found [80]. Surface conditioning may, therefore, eliminate the necessity of removing aged composite restorations. A variety of methods have been used for that purpose, with different results. Air abrasion methods followed by silanization and bonding or by using active MDP-based monomers can be used [81]. However, silica coating followed by silanization seems to deliver significantly higher BS values (46–52 MPa) than specimens treated with phosphoric acid and adhesive only (16–25 MPa) or with HF acid [82]. Silica coating has been shown to provide a reasonably high BS after thermocycling compared to the results obtained with all other methods of treatment [83].

### 7.6. Pre-cementation Shade Verification

Similar to any indirect restoration, PLVs must be tried to minimize the chances of errors after the final cementation. As the color is lighter on dry tooth surfaces, a damp proof try-in should be performed. The try-in pastes supplied by the manufacturers can ensure this wet simulation, in addition to mimicking the color of resin cement. However, great care should be taken to eliminate it. One method of elimination from the intaglio ceramic surface is the application of 37% phosphoric acid, used only for cleaning and not for etching, followed by rinsing with water and an ultrasonic bath. If incompletely eliminated, the quality of bonding is compromised, and a change in color at the interface is expected [4]. Products based on sodium hydroxide can effectively remove various contaminants from the ceramic surface (e.g., grease and oils from handling, surfactants from acid gels, saliva, etc.) and provide a clean surface for resin bonding. They can also be used after HF etching [84].

### 7.7. Cementation and Curing Procedures

Whether to polymerize the DBA prior to cementation or not remains debatable. The unpolymerized dentin–resin hybrid layer might collapse due to pressure during the seating of the restoration. However, if DBA is pre-polymerized, concerns related to its thickness exist. Film thickness depends on the type of DBA: it ranges from 0 to 500 µm. The topography of the tooth preparation can also affect DBA thickness: it ranges from 60 to 80 µm on a smooth convex surface and to 200–300 µm on concave structures, such as marginal chamfers, hence interfering with the complete seating of the restoration. Unless the clinician uses DBA with extremely thin films, it is, therefore, recommended that the DBA be kept unpolymerized. However, two problems emerge during the seating of the restoration and can subsequently compromise the quality of bonding: (a) the outward flow of dentinal fluid dilutes the bonding agent and hinders the penetration of the resin into the microporosities; and (b) the pressure of the luting resin can collapse the demineralized dentin. Facing this dilemma, immediate dentine sealing has been advocated since 1996. If already implemented, pre-polymerization of DBA is not indicated, as it would prevent the complete seating of the restoration [85]. 

Cement thickness is one factor that influences the shear BS of luted veneers. Chun et al. (2010) [32] stated that thermal stress causes compressive and tensile forces in the ceramic and induces cracks when the luting cement layer is thick relative to the ceramic layer. Many factors govern cement thickness, one of them being the cement space provided either digitally or by the die spacer thickness and the number of coats applied. When more than 40.55 ± 12 µm, it leads to a decrease in BS values, particularly during thermal cycling [86,87]. However, the final cement thickness is related rather to the degree of internal fit than to the thickness of the die spacer. The lab fabrication procedure, the material of the veneer, the geometry of the preparation, the seating technique, and the viscosity of the resin cement are all factors affecting the ceramic/composite ratio of thicknesses (CER/CPR). A high ratio (>3) favors a homogeneous stress distribution in the laminate. It is explained by a lower polymerization shrinkage of the resin cement and the mismatch in the coefficient of thermal expansion of the restorative material. This ideal post-cementation situation is not obtained where porcelain has to be thinned to reproduce a natural contour of the restoration, such as facially and cervically. Unless the restoration is over-contoured, only a good internal fit (around 100 μm) can prevent such a drop in the ratio to below a critical value [88]. 

Clinically, color reproduction poses a challenge because of color interaction and superposition between the veneer, the cement, and the underlying substrate [89,90]. Little information is available about the appropriate cement thickness to improve the color match. However, Niu et al. [91] demonstrated that the color differences observed between different ceramic blocks were related to both the color and thickness of the resin cement. Additionally, an interaction exists between cement’s color and its thickness, even if not all types of cement have their color equally affected by the change in thickness [92].

### 7.8. Shade of Substrate

Ceramic thickness and relative translucency also have an effect on the perceived color of the cement and, ultimately, the final color of the restoration. Azer et al. (2011) found a significant interaction between substrate shades and the bonded ceramic laminate veneer shades, both in lightness and chroma parameters [93]. With a ceramic thickness of 2.0 mm, no detectable color differences were noted. However, when ceramic thickness decreased to 1.5 mm, color differences were noticeable only with color recording devices, whereas with ceramic thickness of less than 1.0 mm, they were detectable by the human eye. Knowing that ceramic veneers are generally fabricated to be 0.3 to 1.0 mm in thickness, the underlying substrate shade has a significant role in influencing the final shade regardless of ceramic shade, particularly with thinner veneers [94]. Lighter and darker substrate colors show more color shifts, especially with thin veneers. Therefore, clinicians need to consider the masking ability of porcelain restorations in managing cases with severe tooth discoloration in terms of thickness and opacity [95].

### 7.9. Resin Cement Shade and Opacity

Resin cement is available in different colors and translucencies. They may contribute to mask darker and discolored substrates [96]. The color change effect of resin cement decreases when the ceramic thickness increases [97]. However, in thin veneer sections, resin cement of high value and low chroma, as well as of high white opacity, can increase the value and reduce the chroma of ceramic veneers, therefore having an effect on the final color of the restoration [98]. The brightness of the veneer increases with any shade but mostly with high opacity and value shades. It decreases with the lowest-value shades. In case the chroma should be increased, a resin cement of high chroma and low value is recommended [98].

With high-translucent ceramic material, chroma and translucency of the resin cement can affect the final color, even in thicker sections. Dede et al. (2016 and 2017) showed that shade A2 HT veneered cores of 1.5 thickness combined with the universal shade of resin cement caused a change toward yellow [99]. The use of a translucent shade of cement shifted the color of ceramics toward blue. Kürklü et al. (2013) demonstrated that the mean color differences in cemented A1 feldspathic porcelain of 0.5 mm and 1.0 mm thickness were below the clinical acceptability threshold only when utilizing a translucent clear cement shade [100]. They concluded that changes in porcelain thickness or cement shade may adversely affect the basic esthetic properties of these materials.

## 8. Optical Attributes of the Ceramic Restoration

### 8.1. Opacity and Translucency

Opacity and translucency are two opposite parameters related to the thickness that adds a different level of complexity to the color-matching process [101]. When restoration thickness is decreased, masking more chromatic and discolored teeth becomes difficult. In lighter and darker substrates, the change in the final color of the restoration relative to the chosen shade of ceramic veneer is significantly higher in thinner ceramic materials (0.3 to 0.5 mm) than in thicker ones (0.7 to 1 mm) [99]. 

Other factors influence the translucency of dental ceramics, such as crystalline structure, grain size, and pigments. With a crystal size smaller than the wavelength of visible light, the ceramic would appear transparent. Larger crystals are responsible for light scattering within the body and diffuse reflection, which make the ceramic appear opaque. Part of the light may be absorbed. Scattering the light occurs via reflections and refractions at the interfaces between phases, such as between adjacent crystals and between crystals and the glass phase. The degree of scattering is a function of the relative refractive indices of the different phases and the particle sizes, shapes, and volume concentrations [5]. Pires et al. (2017) demonstrated that the difference in shade between a ceramic veneer and the final shade of the luted veneer was lower for high-opacity ceramics than for low-translucency ones [95]. Consequently, in cases of discolored substrates, it is recommended to consider increasing the thickness and/(or) opacity of the material.

### 8.2. Masking Properties of the Material

The difference in composition of the ceramic materials used for PLVs influences their optical properties and, more precisely, translucency. Ceramics with 70% volume of LDS crystals have higher masking properties than those with 35–45% volume of leucite crystals and are certainly better than feldspathic materials with 30% volume of crystalline particles, the latter material having the higher translucency and less potential for masking discolorations [102]. Within the LDS products, the degree of opacification can be controlled according to the clinical needs via adapting the LDS crystals content. Increasing crystal concentration, such as in LT materials, increases light scattering and consequently imparts restorations with higher masking properties compared to HT or feldspathic ceramics [102]. Lee et al. (2015) observed that the level of translucency is controlled by the ratio of large to small crystals [5]. While high-translucent (HT) ceramics have a small number of large crystals, low-translucent (LT) ceramics contain a large number of smaller crystals.

## 9. Polymerization of the Resin Cement

### 9.1. Polymerization Mode of Resin Cement

Resin-based cement used to bond PLVs is either light-activated (LA) or dual-cured (DC). Selection depends on the opacity and thickness of the ceramic (see Table 5), making to color stability of the luting resin an important consideration. Some DC systems perform a noticeable color change as a result of the tendency of aromatic and aliphatic tertiary amine co-initiators used in the polymerization process to readily oxidize to form colored oxidized products. A further potential source of color change is 2-Hydroxyethyl methacrylate (HEMA) within the resin used to provide more hydrophilic properties to the resin. HEMA also makes the resin prone to water sorption leading to a reduction in cross-linking of the cure and can, accordingly, weaken the cement [103,104]. HEMA-free (e.g., OptiBond All-In-One; Kerr Co, Orange, CA, USA; AdheSe One F; Ivoclar Vivadent, Schaan, Liechtenstein) and non-TEGDMA (Triethylene Glycol Dimethacrylate) (eg. Maxcem Elite Universal Resin cement; Kerr Corp., Orange, CA, USA) containing resin cement, such as with some self-adhesive products, have shown higher color stability [105].

Camphorquinone (CQ) itself, the most widely used photo-initiator, has an intense yellowish color. It might also be a source of color change once photo-polymerized. Depending on the product, either photo-bleaching occurs, or its color is maintained after activation while shift occurs in the other components [106,107]. In recent years, new photo-initiators have replaced CQ in some new brands of LC and DC resin cement, such as phenyl propanedione (1-phenyl-1,2-propanedione). A more light-sensitive initiator—Lucirin TPO, and derivatives of dibenzoyl germanium, such as Ivocerin—do not need additional co-initiators because it decomposes directly into one or more free radicals upon receiving sufficient energy at the correct wavelength. They are completely colorless after photo-polymerization, and their resin polymers are less yellow [108,109,110].

In general, inadequate polymerization with a reduced degree of conversion may cause changes in the physical characteristics of resin-based components, affecting their mechanical properties, altering dimensional stability, and decreasing bonding to tooth structures, capable of resulting in the unsatisfactory clinical performance of these materials. Any delay in the photo-polymerization procedure of any type of resin cement results in a decreased degree of conversion and, therefore, in higher water sorption of internal and external fluids, which is one cause of instability of the color of resin cement [99]. Dual polymerized cement that does not cure properly may result in adverse chemical reactions and permeability issues affecting the esthetic properties of the material, which will be prone to discoloration due to oral fluids and gingival bleeding. A further limitation of the use of DC resin cement is the incompatibility with some self-etch adhesives, known as acidic simplified adhesives. The oxygen-inhibited layer of the simplified adhesives reacts with the tertiary amines of dual-cured cement, leading to an incomplete set and poor bonding. The acidic monomers can also create a hypertonic environment that draws fluids osmotically from the bonded hydrated dentin through the permeable adhesive layer [111,112].

Photo-polymerization units available on the market vary in light intensity and speed, imparting a crucial effect on BS to the underlying substrate. The use of high-intensity light (e.g., plasma arc light units) compared to halogen light might induce higher polymerization stresses at the interface between porcelain and adhesive on one side and between adhesive and tooth substrate on the other side. This suggests that rapid photo-polymerization may cause considerable shrinkage (see Table 6) [113]. 

### 9.2. Post-Cementation Risk Factors

The cementation procedure is not limited to a proper fixation of the veneer on the preparations via a judiciously selected resin cement. It also includes post-cementation finishing, occlusal adjustment, and polishing, followed by home care maintenance instructions and a recall program. 

The first aim of post-cementation finishing is to eliminate any residual cement at the margins of the restoration that might impair the health of the gingival tissues. Considering that fully set resin cement is extremely difficult to break, it is recommended to first tack cure the excess cement for 2–3 s at a 1–2 cm distance in order to obtain a gel-like extruded material, easy to break with a probe or sickle-shaped instrument [4,114]. During the following final polymerization, omitting the application of glycerin-based paste at restoration margins might lead to the formation of an oxygen-inhibited layer with a lower polymerization level [115].

Many geometric-mechanical risk factors can be responsible for immediate or time-related post-cementation mishaps, such as structural loss and/or debonding of the PLV. Fractures can occur if adequate space is not allowed for the ceramic material, i.e., insufficient preparation. However, in patients with bruxism, fracture events increase considerably despite enough thicknesses. The probability of debonding is almost three times higher [116]. It can be extrapolated from this finding that any overloading conditions, such as resulting from unevenly distributed occlusal contacts and/or traumatic anterior guidance, might lead to mechanical failures of the restorations (chipping or fractures), particularly if the veneer is well-bonded [117]. The same is true with direct trauma. Conversely, adhesive fractures generally occur if a bonding problem exists 116. It is, therefore, crucial to operate an immediate occlusal adjustment and recommend a mouthguard for patients with parafunctional habits or involved in contact sports. They must also be instructed to avoid hard foods, chewing on ice, nail-biting, and generating any sort of micro-trauma and overload. 

It is essential to mention that leaving a preparation with sharp line angles can generate internal microcracks that propagate in relation to time-dependent stresses or fatigue. While chipping of a part of the PLV occurs if it has not been properly adhered, the internal stress can initiate a crack line if the PLV is strongly bonded to the tooth [31,118,119]. Finally, any post-cementation correction must be performed under a cooling spray with fine and microfine diamond finishing burs, microfine silicone points, 30-blade finishing burs, and polishing discs and strips [113].

## 10. Challenges and Trends

Many factors contribute to the success of PLV treatment, among which is the conservation of enamel via adequate control of the preparation depth. In this respect, tooth reduction guides might be useful to provide more accuracy for tooth preparation procedures [120]. From this point on, the selection of the restorative material relies on the type of supporting tooth structure, the volume to restore, the architecture of the preparation, and the number of occlusal stresses to anticipate.

Based on the treatment goal of being as conservative and esthetic as possible, the first choice still lies with either feldspathic porcelain or glass-matrix ceramics in high-stress cases with reduced enamel support and where large volumes have to be restored. They showed long-term survival rates of about 80.1–100% in less than 5 years [28]. Conversely, while heat-processed composite resin restorations still suffer from limited longevity, as they remain susceptible to discoloration, wear, and marginal fractures [27], indirect hybrid ceramics have a reduced optical matching capacity of the neighboring structures.

The outcome of the fabrication process is directly related to the accuracy of the impression and laboratory work. The digital scanning of the preparation(s) and a computer numerical control generation of both casts and restorations permit easier fabrication and a net improvement of veneer fit. Additive manufacturing allows for forming of complex shapes with hollow interiors and the reduction in material waste. However, when compared to subtractive procedures, results related to accuracy are still conflictual. Further search is needed for the best materials to be used by the 3D printer to make the final prosthesis, the shape of the margins, and printer hardware [121].

On the other hand, as adhesive bonding is considered to be a challenging procedure, multiple risks coexist, particularly when cementing multiple ceramic veneers: dislodgement, rotation or misfit of the restoration, and/or serious difficulty in removing the excess cement while controlling pressure in the correct seating position without the risk of fracturing the ceramic material. Fabricating a custom 3D printed guide for veneer bonding can provide significant assistance to this complex and stressful clinical procedure [122]. However, reducing the polymerization stresses at the tooth-restoration interface is crucial. Efficient and controlled photocuring is necessary, a procedure that must be adapted to the clinical situation in terms of the type of restorative material, thickness, shade, relative translucency, geometry, and distance from the tip [113]. When the ceramic/composite ratio of thickness is high, because large volumes of ceramic material are needed to optimize teeth alignment, orthodontic correction must be considered prior to veneer treatment [121].

## 11. Conclusions

Since the introduction of etched porcelain veneer restorations almost 40 years ago, indicated for solving esthetic and/or functional problems in the anterior dentition, advances in adhesive technologies and ceramics have proven that porcelain veneers are durable and esthetic treatment modalities. These past years of success can be attributed to great attention to detail in each area of execution and, in particular, the adhesive cementation procedure. A suboptimal technique can be a source of complications, whether esthetic, mechanical, or biological. However, clinicians must understand that risk factors affecting the quality of cementation might stem from every step performed prior to cementation—case selection and treatment planning, tooth preparation, impression, and adequate choice of the restorative material. Due to the thin nature of such restorations, any shift from well-established standards has an esthetic and biomechanical consequence and might also affect the adhesion quality. Scientific evidence guides the careful execution of this process to achieve predictable outcomes.

## Figures and Tables

**Table 1:** Composition, mechanical, and physical properties of some ceramic-based materials used for PLVs.

Material	Brand (Manufacturer)	Composition	Translucency Parameter	Average Crystal Size	Fracture Toughness (MPa.m^0.5^)	Elastic Modulus (E) in GPa
Feldspathic ceramic	Vitablocs Mark II (Vita Zahnfabrik, Bad Sackingen, Germany)	56–64% SiO_2_, 20–23% Al_2_O_3_, 6–9% Na_2_O, 6–8% K_2_O	29.0 ± 0.7	±15 µm	0.84 ± 0.06	±45
Lithium disilicate ceramic (LT)	IPS e.max Press or CAD (Ivoclar Vivadent, Schaan, Liechtenstein)	58–80% SiO_2_, 11–19% Li_2_O, 0–13% K_2_O, 0–8% ZrO_2_, 0–5% Al_2_O_3_	26.0 ± 0.6	±1.5 um	1.23 ± 0.26	±95
Zirconia-reinforced glass–ceramic (HT)	Vita Suprinity (Vita Zahnfabrik, Bad Sackingen, Germany)	56–64% SiO_2_, 1–4% Al_2_O_3_, 15–21% Li_2_O, 8–12% ZrO_2_, 1–4% K_2_O	31.0 ±1.0	±0.5 µm	1.25 ± 0.79	±70
Yttria-stabilized tetragonal zirconia polycrystal (HT)	VITA YZ (Vita Zahnfabrik, Bad Sackingen, Germany)	90.9_94.5% ZrO_2_, 4 6% Y_2_O_3_, 1.5–2.5% HfO_2_, 0–0.3% Al_3_O_3_, 0–0.3% Fe_2_O_3_	14.44 ± 0.34	±350–500 nm	±4.5	±210

**Table 2:** Different protocols of intaglio surface treatment according to type of ceramic, acidic concentration, and silane conditioning.

Ceramic Type	Conditioning	Rinsing Time	Silanization Time
Feldspathic [3]	9.5% HF for 1 to 2.5 min	1 min	1 to 5 min depending on silane system, in one or 2 bottles
Leucite-reinforced [3]	9.5% HF for 60 s		
Lithium disilicate-reinforced [3]	9.5% HF for 20 s		

**Table 3:** Types of ceramic primers.

Presentation and Recommendations of Use	One Bottle Silane [70]	Two Bottles Silane [75]
Bottle content	1% to 5% silane in a water/ethanol solution with an acetic acid adjusted pH of 4 to 5.	Container1: unhydrolyzed silane/ethanol solution. Container 2: an acetic acid/water solution.
Shelf life	Limited to one year	Around two years
Recommendation of use	Refrigerated storage Bringing refrigerated containers to room temperature prior to use Discard when solution appears cloudy or milky or any type of precipitate is noticed	Must be discarded after shelf-life is reached.
Number of coats	Maximum 2 coats	
Time for hydrolysis to occur	Already pre-hydrolyzed	From 0 to 5 min
Effect of treatment	Silane in the solution reacts with the substrate, forming chemical bonds	The unhydrolyzed silane/ethanol solution in container 1 serves as the primary active component. The acetic acid/water solution in container 2 helps facilitate the hydrolysis of the silane. When the contents of both containers are mixed, hydrolysis occurs

**Table 4:** The 3 silane layers formed after silane application and methods of elimination of the two undesirable outermost layers.

Layers	Composition	Method of Elimination	First Alternative Method of Elimination	Second Alternative Method of Elimination
Outermost layer [67]	Small oligomers	Washed away by organic solvents or water at room temperature	Apply the silane followed by hot air drying (50 ± 5 °C) for 15 s for proper solvent evaporation. Then rinse with hot water (80 °C) for 15 s followed by another hot air drying for 15 s.	Try-in step performed following the silanation
Middle layer [76,77,78]	Hydrolyzable oligomers could compromise the coupling of the cement	Removed with hot water		
Inner layer [76,77,78]	Monolayer covalently bonded to the silica phase of the ceramic and is hydrolytically stable	Not to be removed	N.A	N.A

**Table 5:** Classification of resin cement with incurred risks, according to polymerization mode.

Resin Cement	Indications	Contra-Indications	Risk
Light-cured [104]	Thin and translucent veneers	Thick and more opaque veneers	Incomplete photo-polymerization at the thick parts of the veneer
Dual-cured [103]	Thick and more opaque veneers	Thin HT veneers	Discoloration, a prejudice in thin veneers cases
Base paste of the dual-cured system [103]	Optional with some dual-cured resin cement	Thick and more opaque veneers	Sub-optimal cure of the resin cement

**Table 6:** Factors affecting polymerization of resin cement.

Factors	Clinical and Material Parameters	Clinical Protocols	Risks
	Thickness of restoration	Thickness should be ≤0.8 mm for purely LC cement and ≤2.0 mm for DC cement	Cement hardness and efficiency of polymerization decreases with thickness
	Translucency/Opacity of restoration	Greater degree of polymerization with more translucent ceramics, such as feldspathic porcelains and LDS HT.	Longer photoactivation time with opaque porcelains (twice as long)
		Shade has less effect on polymerization than translucency	Longer photoactivation time with darker restoration shade (up to double)
Factors related to resin cement [113]	Mode of polymerization (LC or DC)	DC cement should be light-cured to gain initial immediate set. Protect cement on the margin.	Lower degree of conversion with delayed photo-activation and/or leaving cement margin exposed to ambient oxygen.
	Opacity of cement	Increase photoactivation time for opaque cement.	Shorter photoactivation time affects quality of cement.
	Film thickness	Longer photoactivation time is required with film thickness of >40 μm	Type II cement or less than optimal internal fit require longer photactivation time
	Filler content and particle size	High filler content and particle size improve depth of cure.	When flowable composites are used for cementation, less depth of cure is expected due to very small filler particle size.
Factors related to photo-polymerization units (PPU) [113]	Distance	Light tip as close as possible to the veneer surface.	Photo-polymerization time should be increased with distance.
	Intensity of light	No less than 800 mw/cm^2^	Debris buildup, marring, or discoloration of the wand tip; Aging of the light bulbs and its reflector or blistering and cracking of the filter; Damage because of sterilization; Mismatch between light polymerization tip and radiometer aperture.
	Photo-polymerization protocols	Conventional protocol or soft start polymerization (ramped or stepped)	Conventional protocol with high intensity PPUs generates “stress accumulation” due to polymerization shrinkage at resin/dentin interface.
	Rate of photo-polymerization	Slow rate (40–60 s) with halogen light (low intensity 800 mW/cm^2^) versus rapid rate (3–6 s) with PAC lights.	Rapid rate may cause excessive polymerization shrinkage.
	Duration of exposure	15–20 s with high intensity PPUs (>1000 mW/cm^2^). Longer (×2 manufacturer’s instructions) for opaque ceramics and cement, darker shade of restoration, and increased distance.	Suboptimal polymerization if duration is not respected (with PAC, duration must be more than recommended by manufacturer)
	Radiation Wavelength	Light WL should be within range of activation of the photoinitiator (from 420 to 500 nm with CQ)	Otherwise no initiation of polymerization. Only temperature elevation occurs.

Lithium disilicate high translucency = LDS HT; Resin cement = RC; Light-cured = LC; Dual-cured = DC; Camphorquinone = CQ; Wavelength = WL; Photo-polymerizing unit = PPU; Plasma arc curing = PAC.

## Data Availability

Not applicable.
